# Tetanus vaccine during pregnancy: data of a tertiary hospital in Turkey

**DOI:** 10.3906/sag-2001-77

**Published:** 2020-12-17

**Authors:** Gülşah DAĞDEVİREN, Gökçen ÖRGÜL, Aykan YÜCEL, Dilek ŞAHİN

**Affiliations:** 1 Department of Perinatology, Etlik Zübeyde Hanım Women’s Health Care, Training and Research Hospital,University of Health Sciences, Ankara Turkey

**Keywords:** Maternal tetanus, tetanus vaccine, pregnancy

## Abstract

**Background/aim:**

To evaluate the prevalence of tetanus vaccination in pregnant women and determine the factors affecting the vaccination and barriers to vaccination.

**Materials and methods:**

An observational-descriptive study was conducted on 494 women who gave birth at the Etlik Zübeyde Hanım Women’s Health Training and Research Hospital, Ankara, Turkey. Participants were divided into 2 groups, vaccinated and unvaccinated. Sociodemographic characteristics, obstetric history, and prenatal care status were compared between the 2 groups.

**Results:**

There were 242 (48.9%) and 252 (51.1%) women in the vaccinated and unvaccinated groups, respectively. The vaccination rate decreased as the number of pregnancies increased (P = 0.009). As the level of income increased, there was a statistically significant increase in the vaccination rate (P = 0.048). The status of education and having an occupation did not affect the vaccination rate (P > 0.05). The vaccination rate was higher in women with regular follow-ups when compared to those who did not get a regular follow-up (76.5% vs. 38.7%) (P = 0.001). The vaccination rate was significantly higher in women who had knowledge about tetanus vaccine during pregnancy (P < 0.005).

**Conclusions:**

All pregnant women should be encouraged to get regular antenatal care to increase vaccination rates. Health care providers should give all pregnant women detailed information about the safety, effectivity, and benefits of vaccines.

## 1. Introduction

Tetanus is caused by contamination of *Clostridium tetani* spores into wounds after the disruption of skin integrity and mucous membranes [1]. Tetanus that occurs in women during pregnancy or within 6 weeks after delivery is defined as maternal tetanus (MT). Neonatal tetanus (NT) is a disease that occurs in newborns due to childbirth under poor hygiene conditions. NT is an important cause of perinatal morbidity and mortality. NT is common in developing countries where maternal immunization is insufficient, especially when delivery is not performed under sterile conditions. Up to 100% of untreated cases result in death [2].

Tetanus is a disease that can be prevented by vaccines containing tetanus-toxoids. Antibodies produced by maternal immunization that pass to the fetus via the placenta protect the baby in terms of tetanus during the neonatal period [2]. Elimination of maternal NT (MNT) has been defined as less than 1 NT case per 1000 live births in a specific region by the World Health Organization (WHO). MNT elimination strategies include vaccination, hygienic practices at delivery, and surveillance 1World Health Organization (2000). Maternal and neonatal tetanus elimination by 2005: Strategies for achieving and maintaining elimination [online]. Website https://www.who.int/vaccines-documents/ [accessed 16.12.2018].
.

The WHO recommends combination vaccines, including tetanus toxoid and diphtheria toxoid (Td), for immunization against tetanus 2World Health Organization (2018). Immunization, vaccine and biological [online]. Website https://www.who.int/immunization/diseases /tetanus/en/ [accessed 16.12.2018].
. The first dose is recommended at the first prenatal visit and the second dose is recommended 4 weeks following that for pregnant women whose vaccination status is unknown or those who have not previously been vaccinated. Vaccine doses should be completed at least 2 weeks prior to delivery to ensure adequate protection after the second dose of the vaccine. At least 6 months after the second dose, the third dose should be completed and then, 1 additional dose should be administered with 1-year interval 3World Health Organization (2019). Protecting All Against Tetanus [online]. Website https://www.who.int/immunization/diseases/tetanus/Protecting_All_Against_Tetanus [accessed 10.12.2019].
.

According to the 2018 WHO data, the Td vaccination rate of 2 doses in pregnant women was 72% worldwide, and this rate was about 55% in Turkey 4World Health Organization (2019). Immunization, Vaccines and Biologicals [online]. Website https://www.who.int/immunization_monitoring/globalsummary/timeseries/tsco veragett2plus [accessed 05.07.2019].
. It is recommended that at least 80% of pregnant women should be fully vaccinated against tetanus to achieve and maintain MNT elimination [3]. Td vaccination rates differ among countries such as Thailand 96%, Malaysia 93%, Tunisia 90%, Viet Nam 88%, Spain 84%, and India 81%, where vaccination rates are high and MNT elimination has been achieved 5World Health Organization (2019). Immunization, Vaccines and Biologicals [online]. Website https://www.who.int/immunization_monitoring/globalsummary/timeseries/tsco veragett2plus [accessed 05.07.2019].
.

In this study, it was aimed to evaluate the prevalence of tetanus vaccination in pregnant women who gave birth at the Etlik Zübeyde Hanım Women’s Health Training and Research Hospital, in Ankara, Turkey. Also investigated were the factors affecting vaccination and barriers to vaccination, to understand future needs towards increasing vaccination rates.

## 2. Materials and methods

An observational-descriptive study was conducted with 494 women who gave birth at the Etlik Zübeyde Hanım Women’s Health Training and Research Hospital, in Ankara, Turkey, between January 1, 2019 and January 31, 2019. Signed informed consent was obtained from participants who agreed to be involved in the study. The study protocol was approved by the Ethical Committee of the University of Health Sciences Turkey, Etlik Zübeyde Hanim Women’s Health Training and Research Hospital (02.01.2019-29/1), and complied with the Helsinki Declaration.

A data collection form related to tetanus vaccine was applied via face-to-face interviews by the same investigator (GD). The women were asked various questions regarding their age, number of pregnancies, monthly income, frequency of antenatal care, and whether they had vaccination in this pregnancy. The main refusal reasons were also requested from the women who had not been vaccinated during pregnancy.

In terms of vaccination, the participants were divided into 2 groups, vaccinated and unvaccinated. Women who had received 2 doses of vaccine during their current pregnancy were accepted as fully vaccinated. In addition, women who stated that they had received 2 doses of vaccine in their previous pregnancies and who received a single dose of vaccine in the current pregnancy were also considered to be fully vaccinated. The remaining women were classified as unvaccinated. Sociodemographic characteristics, obstetric history, and prenatal care status were compared between the 2 groups. Factors affecting the decisions of women who did not receive the vaccine were also analyzed.

Data were analyzed using descriptive statistics, such as the mean, standard deviation, and frequency using IBM SPSS Statistics for Windows 23.0 (IBM Corp., Armonk, NY, USA). Variables in the groups were evaluated using the Shapiro–Wilk test for distribution. Normal distributions were evaluated using parametric tests (independent t test) and those without normal distribution were evaluated using nonparametric tests (Mann–Whitney U test). The chi square test was used to evaluate the categorical variables.

## 3. Results

Among the 494 women who participated in the study, 74.1% (n = 366) stated that they had been vaccinated in their last pregnancy and the remaining 25.9% (n = 128) said they had never been vaccinated. Of the women who received the vaccine, 45.3% (n = 224) had been vaccinated with 2 doses and 28.7% (n = 142) had been vaccinated only with a single dose. Among the women who received a single dose of vaccine, 18 were considered to be fully vaccinated because they had received 2 doses of vaccine in their previous pregnancies. As a result, the rate of vaccinated and unvaccinated women was 48.9% (n = 242) and 51.1% (252), respectively. The Figure shows the groups in detail.

**Figure:**
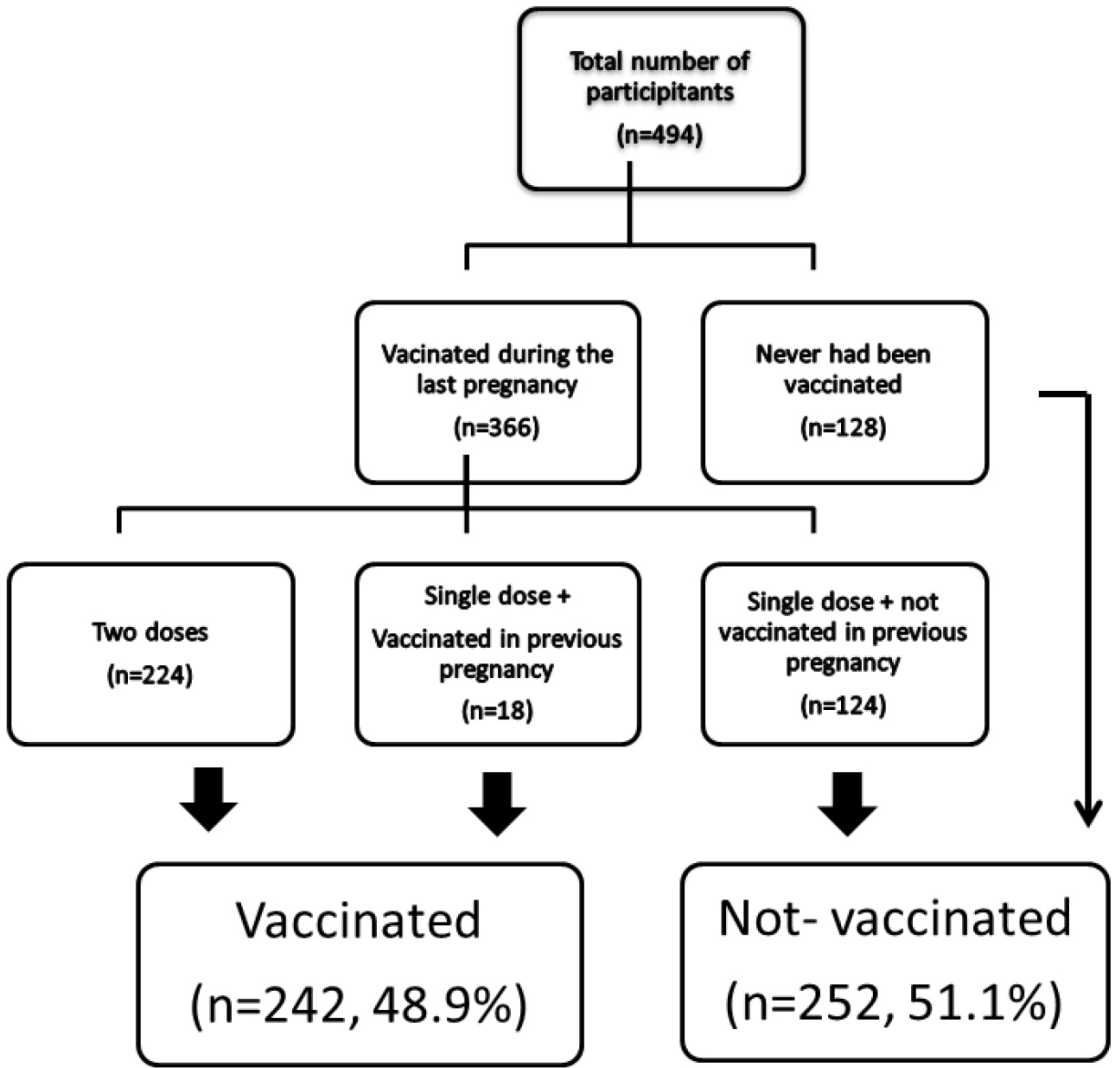
Flowchart of the study.

Table 1 shows the comparison of the demographic and socioeconomic characteristics of the women between the 2 groups. The vaccination rate was 82.1% and 64% in the nulliparous and multiparous women, respectively. The vaccination rate decreased as the number of pregnancies increased (P = 0.009). As the level of income increased, there was a statistically significant increase in the vaccination rate (P = 0.048). The status of education and having an occupation did not affect the vaccination rate (P > 0.05). As shown in Table 2, it was observed that antenatal follow-up frequency had an impact on the rates of vaccination. The vaccination rate was higher in women with regular follow-ups when compared to those who did not get a regular follow-up (76.5% vs. 38.7%) (P = 0.001).

**Table 1:** Comparison of the demographic and socioeconomic characteristics between the fully vaccinated and unvaccinated pregnant women.

	Vaccinated n = 366 (74.1%)	Unvaccinated n = 128 (25.9%)	P-value
Age	28.49 ± 5.94	29.23 ± 6.11	0.232†
Gestational age at birth	38.13 ± 2.59	37.78 ± 3.16	0.542†
Parite			0.221*
Nullipar (n = 162)	162 (36%)	30 (23.6%)	
Multipar (n = 332)	235 (64%)	97 (76.4%)	
Education			0.311*
Illiterate (n = 21)	10 (47.6%)	11 (52.4%)	
Primary school (n = 234)	174 (74.4%)	60 (25.6%)	
Secondary school (n = 167)	128 (76.6%)	39 (23.4%)	
University or higher (n = 72)	54 (75.0%)	18 (25.0%)	
Socioeconomic status			0.048*
Low (n = 274)	196 (71.5%)	78 (28.5%)	
Moderate (n = 185)	137 (74.1%)	48 (25.9%)	
High (n = 35)	33 (94.3%)	2 (5.7%)	
Occupation			0.829*
Housewife (n = 406)	300 (82.0%)	106 (28.0%)	
Working (n = 88)	66 (75.0 %)	22 (25.0%)	
Smoking			0.876*
No (n = 438)	325 (65.2%)	113 (34.8%)	
Yes (n=56)	41 (53.3%)	15 (46.7%)	

*Chi square test; † Independent t test.

**Table 2:** Characteristics of the antenatal follow-up of the vaccinated and unvaccinated women.

	Vaccinatedn = 366 (74.1%)	Unvaccinatedn = 128 (25.9%)	P-value
Regular antenatal care			<0.05*
Yes (n = 463)	354 (76.5%)	109 (23.5%)	
No (n = 31)	12 (38.7%)	19 (61.3%)	
Type of prenatal care facility			<0.05*
Family health center (n = 9)	8 (88.9%)	1 (11.1%)	
State hospital (n = 423)	323 (76.4%)	100 (23.6%)	
Private practice (n = 32)	22 (68.7%)	10 (31.3%)	
University hospital (n = 12)	10 (83.3%)	2 (16.7%)	
Absence of prenatal care (n = 18)	3 (16.7%)	15 (83.3%)	
Informed about tetanus vaccine			<0.05*
Yes (n = 340)	305 (89.7%)	35 (10.3%)	
No (n = 154)	61(39.6%)	93(60.4%)	

* Chi square test.

The total number of women who stated that they were informed about Td vaccine during pregnancy was 340 (68.8%), and the rate of vaccination in this group was 89.7%. Moreover, 212 (62.3%) of the women were informed by doctors, 123 (35.8%) by midwives/nurses, and the remaining 6 (1.9%) via media/internet. The vaccination rate was significantly higher in women who had knowledge about tetanus vaccine during pregnancy than in those who did not know about the vaccine (P < 0.005).

Table 3 shows the detailed data of the unvaccinated women. Of the 128 women who were unvaccinated, 46.8% (n = 60) stated they did not know that they should have been vaccinated, 12.5% (n = 16) thought that the vaccine was not useful, and 11.7% (n = 15) said that they did not know where or when to get the vaccine. Moreover, 10.9% (n = 14) had not been vaccinated because they had been vaccinated during their previous pregnancy.

**Table 3:** Main reasons for vaccine refusal.

	n	%
I did not know I had to be vaccinated	60	46.8
I do not think the vaccine is useful	16	12.5
I did not know where or when to get the vaccine	15	11.7
I was already vaccinated in a previous pregnancy	14	10.9
I did not have time to get the vaccine	11	8.5
My obstetrician did not recommend it	5	3.9
I’m afraid of the needle	3	2.3
I have concerns about the contents of the vaccine	3	2.3
I thought the vaccine would have adverse effects on the baby	1	0.7

## 4. Discussion

Tetanus occurs as a result of the contamination of wounds with *Clostridium tetani* spores in women without protective antibodies. It is not possible to eradicate the disease because these spores are widespread in the soil and feces of animals. Therefore, it is important to ensure surveillance by recommended vaccination programs 6World Health Organization (2000). Maternal and neonatal tetanus elimination by 2005: Strategies for achieving and maintaining elimination [online]. Website https://www.who.int/vaccines-documents/ [accessed 16.12.2018].
.

Immunization with tetanus vaccine during pregnancy is an effective method to protect the newborn against disease. Tetanus vaccines are available as toxoid alone (TT), with diphtheria toxoid (Td), or in combination with diphtheria and pertusis vaccines (Tdap). A single dose of Tdap vaccine is recommended for pregnant women in the USA within 27–36 weeks of gestation in every pregnancy [4]. The WHO recommends using Td for immunization against tetanus [3]. In Turkey, Td vaccine began to be used instead of TT vaccine after August 2004 as a result of the recommendation of the Ministry of Health with the decision of the Immunization Advisory Board 7Ministry of Health of Turkish Republic (2006). Maternal and neonatal tetanus elimination programme guideline [online]. Website http://www.saglik.gov.tr/ extras/birimler/temel/TAG_saha_rehberi.pdf. [accessed 05.07.2019].
. According to the WHO MNT elimination strategies, it has also been aimed in Turkey to reduce the number of NT cases in each region to <1 in 1000 live births and ensure that MT is eliminated. Hence, tetanus vaccines are administered free of charge to all pregnant women in primary health care centers7. The Turkish Ministry of Health has recommended that all pregnant women who had not been vaccinated in childhood, whose vaccination status was unknown, who have not been fully vaccinated, or who have not received a rapel dose in the last 10 years should be administrated a minimum of 2 doses of Td at 4-week intervals during pregnancy. Moreover, a total of 5 doses should be given to all women of reproductive age (15–49 years) 8Ministry of Health of Turkish Republic (2011). Circular letter of expanded immunization programme [online]. Website http://www.saglik.gov.tr/TR/dosya/1-33203/h/gbpgenelge2008.pdf. [accessed 12.11.2011].
.

From 1999 to March 2019, a reduction of about 96% in NT-related deaths was observed through the efforts of the United Nations Children’s Fund, WHO and the United Nations Population Fund 9World Health Organization (2017). Maternal and neonatal tetanus elimination [online]. Website https://www.who.int/ immunization /diseases/MNTE_initiative/en/ [accessed 05.07.2019].
. Despite all of the recommendations and practices, according to the WHO 2018 data, 13 countries have still been unable to achieve MNT elimination 10World Health Organization (2019). Immunization, Vaccines and Biologicals [online]. Website https://www.who.int/immunization_monitoring/globalsummary/timeseries/tsco veragett2plus [accessed 05.07.2019].
. Overall, the tetanus vaccination rate in pregnant women has been reported as 72% according to the WHO November 2018 data10.

In Turkey, MNT elimination targets were achieved in 2009 and this was approved by the WHO. There was only 1 reported NT case in 2014, and there have been no NT cases reported in the last 5 years in Turkey. According to the 2019 WHO data, the rate of Td vaccination during childhood was 96% while the rate was only 55% during pregnancy in Turkey10. Unfortunately, this rate is far from the intended rate (80%); hence, it is important to take the necessary precautions about this problem. It was demonstrated that the rate of fully vaccinated women was 48.9% in the current study population. This result was similar to the 46.7% vaccination rate obtained by Maral et al. in another study conducted in Ankara [5]. These results showed that the Td vaccination rate was about 50% in women who delivered at tertiary hospitals and these findings may not reflect the real status of national vaccination rates. Thus, MT vaccination might be less than 50% in some regions.

According to the WHO recommendations, in a country where at least 80% of mothers are vaccinated against tetanus, sterile delivery conditions together with hygienic cord care may be sufficient to prevent NT. However, if maternal immunization rates are less than 80%, it is recommended to develop new strategies towards vaccination programs 11World Health Organization (2017). Vaccine-preventable diseases surveillance standards [online]. Website https://www.who.int/immunization/ monitoring_surveillance /burden/ vpd/standards/en/ [accessed 05.07.2019].
. In Nigeria, the Td vaccination rate was reported to be 62% in 2018. Moreover, 89% of births in Nigeria occurred under unhygienic conditions without the aid of skilled healthcare professionals. As a result, 130 NT cases were reported in Nigeria in 2018, which is 1 of the 13 countries where MNT elimination has not be achieved [6]. Similarly, in Pakistan, the vaccination rate during 2018 was 60%, and 52% of the births occurred outside hospitals. Pakistan is also another country where MNT is still an important issue [7]. According to the Turkish national data, the birth rate at a health care centers is 97% 12The Main Report of Turkish Population and Health Survey (2013) [online]. Website http://www.hips.hacettepe.edu.tr/tnsa2013/rapor/TNSA_2013_ana_rapor.pdf [accessed 05.07.2019].
. In this regards, Turkey has shown great success despite the low Td vaccination rate. However, it would still be beneficial to increase tetanus vaccination rates to prevent NT.

The WHO data, together with the current findings, have shown that almost 1 out of every 2 pregnant women in Turkey has still not been vaccinated against tetanus. About 46% of the women who had not been vaccinated in the current study stated that they did not know that they should be vaccinated, and the majority of the remaining cases thought that the vaccine was not useful and they did not know where or when to get the vaccine exactly. There were 109 women who answered no to the question “Did you know that the tetanus-diphtheria vaccine that you got in your pregnancy would protect your baby against these bacteria at birth?”. Among them, 87.2% replied “I would have had the vaccination if I had known”. Unfortunately, these findings have shown that pregnant women are not adequately informed about Td vaccine in Turkey. The main reason for not having enough information is most probably inadequate antenatal care for these women. The rate of vaccination in women with regular antenatal care was 76.5%, whereas only 38.7% of the vaccinated women did not have sufficient antenatal care. Notably, the vaccination rate was 89.7% in women who stated that they had been informed about Td vaccine during pregnancy.

The American College of Obstetricians and Gynecologists put forth some suggestions for all obstetricians regarding maternal vaccination. Obstetricians should know the current vaccine recommendations, inform their patients, and encourage them to vaccinate in order to reduce the incidence of vaccine-preventable diseases [8]. The low rates of tetanus vaccination in pregnancy have been reported to be related with insufficient information provided by obstetricians 13Ministry of Health of Turkish Republic (2006). Maternal and neonatal tetanus elimination programme guideline [online]. Website u2db0 extras/birimler/temel/TAG_saha_rehberi.pdf. [accessed 05.07.2019].
. Controversially, 98.7% of physicians stated that they had recommended tetanus vaccine to their patients in another study [9]. Swamy et al. reported that the rate of vaccination increased if it was recommended by obstetricians [10]. In the current study, 45.3% of the women who had regular antenatal care stated that they were informed by their physicians.

Ensuring hygienic conditions at birth and maternal immunization resulted in MNT elimination in Turkey. On the other hand, the vaccination rate of at least 80% (recommended to prevent NT by the WHO) has not been achieved in Turkey to date. All pregnant women should be encouraged to get regular antenatal care to increase vaccination rates. Health care providers should give detailed information about the safety, effectivity, and benefits of vaccines to all pregnant women.

## Ethics committee approval

This study was approved under number 02.01.2019-29/1.

## Informed Consent

All of the participants signed informed consent forms prior to participation.
